# Comparative Phytochemical Analysis of the Aerial Parts of *Pelargonium radula* and *Geranium macrorrhizum* Cultivated in Bulgaria Using GC-MS and HPLC

**DOI:** 10.3390/ph19030346

**Published:** 2026-02-24

**Authors:** Debora Sabotinova, Petya Boycheva, Nadezhda Ivanova, Velichka Andonova, Vasil Georgiev, Iliya Zhelev

**Affiliations:** 1 Department of Biology, Faculty of Pharmacy, Medical University of Varna, 9000 Varna, Bulgaria; petya.boycheva@mu-varna.bg (P.B.); ilia.slavov@mu-varna.bg (I.Z.); 2 Department of Pharmaceutical Technologies, Faculty of Pharmacy, Medical University of Varna, 9000 Varna, Bulgaria; nadejda.ivanova@mu-varna.bg (N.I.); velichka.andonova@mu-varna.bg (V.A.); 3 Department of Biotechnology, Stephan Angeloff Institute of Microbiology, Bulgarian Academy of Sciences, 4000 Plovdiv, Bulgaria; vasgeorgiev@gmail.com

**Keywords:** *Pelargonium radula*, *Geranium macrorrhizum*, essential oil composition, GC–MS, phenolic acids, flavonoids, HPLC profiling, chemotaxonomy

## Abstract

**Background**: *Geraniaceae* species are widely used in traditional medicine. *Pelargonium radula* and *Geranium macrorrhizum* are aromatic medicinal plants traditionally used in Bulgaria for their antimicrobial, anti-inflammatory, and wound-healing properties. Comparative phytochemical data on *Pelargonium radula* and *Geranium macrorrhizum* cultivated in Bulgaria, however, remain limited. The present work aimed to characterize and compare the chemical composition of essential oils and main phenols, in support of future pharmacological evaluation. **Methods**: Essential oils from aerial parts of both species were obtained by hydrodistillation and analyzed by GC-MS. Through HPLC-UV, ethanol extracts were evaluated to quantify the major phenolic acids and flavonoids. **Results**: The yield of essential oils was 0.10% for *P. radula* and 0.03% for *G. macrorrhizum*, dominated by oxidized monoterpenes, mainly citronellol and geraniol-type compounds. HPLC analysis revealed marked differences in their phenolic profiles. *P. radula* showed a composition with six phenolic acids—primary protocatechuic and ferulic acids, and very low levels of flavonoids, with rutin being the only quantifiable glycoside. In contrast, *G. macrorrhizum* contained nine phenolic acids and four flavonoids, with remarkably high levels of salicylic, rosmarinic, and *p*-coumaric acids, as well as catechins, absent in *P. radula*. **Conclusions**: The two species showed different phytochemical characteristics in both their volatile and non-volatile fractions. *P. radula* is characterized by a citronellol/geraniol-rich essential oil and a moderate phenolic profile, while *G. macrorrhizum* exhibits significantly higher phenolic diversity and abundance. These findings expand the current phytochemical knowledge of both taxa and provide a solid basis for future chemotaxonomic and pharmacological studies. The obtained results suggest that *Geranium macrorrhizum* may be more promising for antioxidant and anti-inflammatory applications, while *Pelargonium radula* may be preferentially explored for ant-microbial purposes.

## 1. Introduction

The *Geraniaceae* family includes over 750 species, mainly belonging to *Pelargonium* and *Geranium* genera, distributed worldwide, and possesses essential economic, ethnopharmacological, and horticultural value [1,2]. While *Pelargonium* species are primarily part of the flora of South Africa, *Geranium* species are widespread across Europe and Asia, with the Balkan Peninsula recognized as a center of biodiversity [3,4,5]. There are several *Geranium* taxa native to Bulgaria. At the same time, species of *Pelargonium* are mainly cultivated not only for their fragrance but also for their therapeutic properties, making them significant in ancient and modern herbal medicine [6,7].

From a phytochemical perspective, the *Geraniaceae* family is characterized by volatile and non-volatile components [1,8,9]. The volatiles define different chemotypes influenced by genetic and ecological factors [1,10,11,12]. The non-volatile fraction mainly comprises phenolics, and the combination of the both groups accounts for the wide range of biological activities associated with the family [13,14,15].

In particular, *Pelargonium* and *Geranium* species have a long history of use in traditional medicine. Infusions, poultices, and decoctions are prepared from these plants, which indicates their long-standing popularity in health practices. *Pelargonium radula* is a perennial aromatic species characterized by densely pubescent leaves with glandular trichomes responsible for essential oil production and a characteristic rose-like fragrance. In contrast, *Geranium macrorrhizum* is a perennial herb with thick rhizomes, palmately lobed leaves, and reddish-pink flowers, commonly occurring in mountainous and forest regions.

For example, species of the genus *Pelargonium* are widely used in local medicine for the treatment of respiratory and gastrointestinal infections [16,17]. Its highly aromatic leaves and essential oil are used in the perfumery and food industries [7], and it is known to have insect-repellent properties [18]. In general, the volatile essential oil extracted from *Pelargonium* species contains high levels of oxygenated monoterpenes, such as citronellol and geraniol. Therefore, it exhibits strong antimicrobial, antioxidant, anti-inflammatory, and analgesic properties [7,18,19,20,21,22,23]. In addition to the volatile fraction, *P. radula* contains phenolic compounds, mainly flavonoids and phenolic acids, which provide additional antioxidant and antimicrobial effects and may act synergistically with the essential oil [22,24]. These compounds are essential to the plant’s traditional use and have potential for modern medical treatments. However, compared to the well-studied *P. graveolens*, *P. radula* remains less studied, despite its similar aromatic profile and biological potential [25].

In addition to their ethnomedicinal uses, *Pelargonium* species are also used in official medicine. The best-documented example is *Pelargonium sidoides*, the root extract of which is the active ingredient in the licensed phytopharmaceutical Umckaloabo^®^, approved in several European countries for the treatment of acute bronchitis and other upper respiratory tract infections [26,27].

*Geranium macrorrhizum* is another important species that belongs to the *Geraniaceae* family and is also deeply rooted in Bulgarian ethnomedicine. It has been traditionally used as an astringent, hemostatic, and tonic, especially for wound healing [28,29,30,31]. Phytochemical studies have shown that the composition of its essential oil varies, with different chemotypes consisting mainly of oxygenated monoterpenes (such as citronellol and geraniol) or sesquiterpene ketones (such as germacrone) [22,32,33]. In addition to its volatile components, *G. macrorrhizum* is also rich in tannins, flavonoids, and phenolic acids, which again contribute to its antioxidant and antimicrobial properties [30,32,34]. All these phenolic constituents, combined with terpenoid-rich oils, offer a wide range of pharmacological effects, including antimicrobial, antioxidant, wound-healing, gastroprotective, and anti-*Helicobacter pylori* effects [30,32,35]. Compared to its close relative, *Geranium robertianum*, *G. macrorrhizum* is less studied, although it is more widely used in Bulgarian traditional medicine [28,36].

Over time, the traditional uses of *Geraniaceae* species have been supported by modern pharmacological studies, mainly focusing on effects related to respiratory, dermatological, and gastrointestinal health [28,37]. In scientific studies, essential oils are particularly recognized for their antimicrobial, insecticidal, and anti-inflammatory properties [1], and phenolic-rich extracts have shown potent antioxidant, anti-*Helicobacter pylori*, and cytoprotective properties [30], thereby complementing and enhancing the biological effects of essential oils [3].

Despite their widespread traditional use and economic importance, comparative phytochemical data on *P. radula* and *G. macrorrhizum* cultivated in Bulgaria remain scarce. Previous studies have focused mainly on volatile compounds, while detailed information on the different extracts is limited [24,38]. As mentioned, these products are exciting because they contain non-volatile secondary metabolites that can enhance or complement the biological activity of essential oils [24,39]. Given the unique ecological and geographical characteristics of the Balkan Peninsula, regional phytochemical studies of these species are of great importance [32].

The selection of *Pelargonium radula* and *Geranium macrorrhizum* for the present study was based on several scientific and practical considerations. Both species are traditionally used in Bulgarian folk medicine and are cultivated under local environmental conditions, making them relevant targets for regional phytochemical investigations. *Pelargonium radula* remains comparatively underexplored in terms of its non-volatile constituents, despite its aromatic similarity to the well-studied *P. graveolens*, whereas *G. macrorrhizum* represents one of the most widely used native *Geranium* species in Bulgaria with documented pharmacological potential. In addition, the two taxa belong to different genera within the *Geraniaceae* family and exhibit distinct chemotaxonomic profiles, providing a suitable model for comparative analysis of volatile and non-volatile metabolites. The limited availability of integrated phytochemical data for these species further justified their selection.

Therefore, this study aimed to characterize and compare the chemical composition of essential oils and to obtain total extracts from *P. radula* and *G. macrorrhizum* herba cultivated in Bulgaria, in preparation for their further pharmacological evaluation. In addition, the study integrates phytochemical, botanical, and ethnopharmacological perspectives to provide a comprehensive comparative evaluation of the two species.

## 2. Results

### 2.1. Essential Oil Yield

Hydrodistillation of the dried aerial parts of *Pelargonium radula* and *Geranium macrorrhizum* was performed using a Clevenger-type apparatus for 3 h, as described in Section 4.2, and yielded essential oils with recoveries of 0.10% and 0.03% (*v*/*w*, relative to dry weight), respectively.

### 2.2. GC-MS Profiling of Essential Oils

The essential oils obtained from *Pelargonium radula* and *Geranium macrorrhizum* were analyzed by GC–MS, and their chemical compositions are presented in Table 1 and Table 2.

The Gas Chromatography–Mass Spectrometry (GC–MS) analysis of the essential oil extracted from the aerial parts of *Pelargonium radula* identified 61 volatile compounds, which accounted for 99.75% of the total ion chromatogram (TIC). All detected constituents were grouped into five structural categories: monoterpene hydrocarbons (MH), oxygenated monoterpenes (MO), sesquiterpene hydrocarbons (SH), oxygenated sesquiterpenes (SO), and a small fraction of non-terpenoid compounds (O). The identified compounds belonged predominantly to monoterpenoid and sesquiterpenoid groups. Oxygenated monoterpenes (MO) made up the most significant portion (85.40%), followed by monoterpene hydrocarbons (MH) (1.94%), sesquiterpene hydrocarbons (SH) (4.88%), oxygenated sesquiterpenes (SO) (6.22%), and non-terpenoid components (1.32%).

The primary components of the oil were citronellol (28.06%) and geraniol (25.05%), along with isomenthone (5.75%), citronellylformate (7.30%), linalool (6.07%), and nerol (1.23%). Other oxygenated monoterpenes detected at lower levels included α-terpineol, menthone, citronellyl acetate, and various monoterpene esters. Sesquiterpenes such as *β*-caryophyllene, *α*-humulene, germacrene D, viridiflorene, and *δ*-cadinene were also present.

A comprehensive qualitative and quantitative list of all compounds was provided in Table 1.

In comparison, the essential oil of Geranium macrorrhizum showed a distinct compositional pattern, as described below. The Gas Chromatography–Mass Spectrometry (GC–MS) analysis of the essential oil extracted from the aerial parts of *Geranium macrorrhizum* identified 51 volatile compounds, accounting for 99.12% of the total ion chromatogram (TIC). The identified constituents were classified into five chemical groups: monoterpene hydrocarbons (MH), oxygenated monoterpenes (MO), sesquiterpene hydrocarbons (SH), oxygenated sesquiterpenes (SO), and other non-terpenoid compounds (O). Oxygenated monoterpenes (MO) were the predominant class, making up 81.25% of the oil, followed by sesquiterpene hydrocarbons (SH) at 14.33%. Minor components included monoterpene hydrocarbons (MH) (1.20%), oxygenated sesquiterpenes (SO) (1.31%), and non-terpenoid compounds (1.02%).

The primary constituents were *β*-citronellol (31.37%), geraniol (14.79%), citronellol (11.54%), linalool (4.03%), citronellylformate (7.55%), and citronellyl pentanoate (4.74%). Other oxygenated monoterpenes detected at lower levels included menthol, iso-menthol, *α*-terpineol, citronellyl acetate, as well as citronellyl propionate, citronellyl pentanoate, geranyl tiglate, and other monoterpene esters derived from citronellol and geraniol.

The sesquiterpene fraction consists of hydrocarbons such as *β*-caryophyllene, *α*-humulene, germacrene D, viridiflorene, *α*-muurolene, *β*-selinene, and cadinene-type compounds (*γ*- and *δ*-cadinene). Oxygenated sesquiterpenes—including spathulenol, cubenol, 1-*epi*-cubenol, *α*-muurolol, and *α*-cadinol were also identified in lower relative abundances compared to the monoterpenoid constituents.

The identified compounds are presented in Table 2.

Additional data are available in the Supplementary Materials (Figures S1 and S2).

### 2.3. HPLC Analysis of Flavonoid and Phenolic Acid Profiles of the Ethanolic Extracts

The phenolic acid and flavonoid profiles of the ethanolic extracts of *Pelargonium radula* and *Geranium macrorrhizum* were evaluated by HPLC, and the results are summarized in Table 3 and Table 4.

High-performance liquid chromatography (HPLC) of the ethanolic extract of *Pelargonium radula* identified a total of six phenolic acids and three flavonoid compounds, with all values expressed as mg/g dry extract (DE) (Table 3). Among the flavonoids, the glycoside rutin was the major constituent (2.51 mg/g DE), while both kaempferol and quercetin were detected below the quantification limit (ULOQ). Hesperidin was found only at trace levels. No flavan-3-ols were detected, as neither (+)-catechin nor (−)-epicatechin was present (NF).

Six phenolic acids were measured in the extract. The most abundant phenolic acids were protocatechuic acid (3.71 mg/g DE) and ferulic acid (3.17 mg/g DE), followed by rosmarinic acid (1.66 mg/g DE) and vanillic acid (1.03 mg/g DE). Lower concentrations were established for gallic acid (0.41 mg/g DE) and caffeic acid (0.17 mg/g DE). Chlorogenic, syringic, and *p*-coumaric acids were either not detected (NF) or were below the quantification limit (ULOQ).

Compared to *P. radula*, the extract of G. macrorrhizum exhibited a more diverse and abundant phenolic profile. The ethanolic extract of *Geranium macrorrhizum* showed a more diverse composition compared to *P. radula*, including nine phenolic acids and four flavonoids (Table 4). All values are given also in mg/g dry extract (DE).

Among the flavonoids, the flavan-3-ols (+)-catechin (2.43 mg/g DE) and (−)-epicatechin (3.17 mg/g DE) were the main representatives. The glycoside rutin was also present (3.08 mg/g DE), while quercetin was measured at 1.23 mg/g DE. Kaempferol was detected but remained below the quantification limit (ULOQ), and hesperidin was not detected.

Nine phenolic acids were identified. The most abundant phenolic acids were salicylic acid (37.33 mg/g DE), rosmarinic acid (18.31 mg/g DE), and *p*-coumaric acid (10.60 mg/g DE). Other phenolic acids composition included vanillic acid (7.64 mg/g DE), syringic acid (3.23 mg/g DE), ferulic acid (2.21 mg/g DE), gallic acid (2.05 mg/g DE), protocatechuic acid (0.77 mg/g DE), and chlorogenic acid (0.96 mg/g DE). Caffeic acid was not found.

Compared to *P. radula*, *G. macrorrhizum* extract exhibited a significantly more phenolic-rich profile, marked by high levels of both hydroxybenzoic and hydroxycinnamic acids, along with various flavonoids, reflecting its greater phytochemical diversity.

Additional data are available in the Supplementary Materials (Figures S3 and S4).

## 3. Discussion

### 3.1. Essential Oil Yield

The essential oil yields obtained from the aerial parts of *Pelargonium radula* (0.10% *v*/*w*) and *Geranium macrorrhizum* (0.03% *v*/*w*) were relatively low, yet fell within the general range reported for members of the *Geraniaceae* family. Essential oil production in *Pelargonium* species is highly variable and depends on numerous factors, including genotype, plant age, environmental conditions, and post-harvest processing. For *P. radula*, the available literature is scarce. However, Gaaffar et al. (2021) reported similarly low recoveries during supercritical CO_2_ extraction of volatiles from *P. radula* leaves, suggesting a naturally modest essential-oil–bearing capacity for this species [40]. Broader comparative data from taxonomically related species further support this trend: *Pelargonium graveolens*, one of the most extensively studied members of the genus, typically yields 0.05–0.30% depending on drying technique, geographic origin, and extraction protocol [41]. These values place our yield of 0.10% for *P. radula* well within the expected range for *Pelargonium* taxa, especially considering that hydrodistillation of air-dried material often produces lower recoveries than fresh distillation or supercritical extraction.

The essential oil yield of *G. macrorrhizum* (0.03%) was even lower, consistent with previously published results. Chalchat et al. (2002) reported extremely modest yields for *G. macrorrhizum* collected in Serbia, often below 0.05% under conventional hydrodistillation [42]. Similar observations were made by Sharopov et al. (2017), who emphasized that *G. macrorrhizum* inherently accumulates volatile constituents in smaller amounts than essential-oil-rich *Pelargonium* species, with yields strongly influenced by ecological and edaphic conditions [32]. Additional comparative data across *Geraniaceae* indicate that *Geranium* oils are generally produced at trace levels, often below 0.1%, and that their bioactive constituents are synthesized through metabolic pathways distinct from those in *Pelargonium* [43].

Overall, the yields observed in the present study align well with the chemotaxonomic patterns of both genera, reflecting inherent physiological differences in glandular trichome density and volatile biosynthetic capacity. The relatively low recoveries for both species are therefore entirely consistent with published evidence. The combined effects of plant genetics, environmental growing conditions, and the use of hydrodistillation on dried botanical material likely influence them.

### 3.2. Essential Oil Composition of Pelargonium radula and Geranium macrorrhizum

The essential oil of *Pelargonium radula* exhibited a chemical profile dominated by oxygenated monoterpenes, particularly citronellol and geraniol, consistent with the citronellol/geraniol chemotype widely reported for this species. Our findings closely align with previous studies demonstrating that *P. radula* essential oil contains high proportions of these two monoterpenoids, along with smaller amounts of linalool, citronellyl esters, and related oxygenated derivatives [44,45]. Seasonal investigations by Kalodera et al. revealed that the relative abundance of these components can vary considerably throughout the vegetative cycle; however, citronellol and geraniol consistently remain the principal constituents across phenological stages [45]. Studies investigating the repellent and biological activities of *P. radula* have associated citronellol-rich profiles with enhanced mosquito-repellent properties and other bioactivities [44,46]. Comparative analyses with closely related *Pelargonium* species, such as *P. graveolens*, further support the predominance of oxygenated monoterpenes as a hallmark of the genus; recent studies confirm that citronellol, geraniol, and their ester derivatives constitute the core of the volatile fraction in these species [47,48]. The biosynthetic basis for this chemotype has been attributed to distinct metabolic pathways regulating monoterpenoid formation in *Pelargonium* populations, as demonstrated by Bergman et al., who identified species-specific patterns in terpene synthase activity driving the accumulation of citronellol- and geraniol-type compounds [49]. Broader phytochemical reviews also emphasize the importance of oxygenated monoterpenes in *Pelargonium* taxa, noting their contribution to antioxidant, antimicrobial, and fragrance-related properties [22,50].

The essential oil of *Geranium macrorrhizum* exhibited a chemical profile dominated by oxygenated monoterpenes, primarily *β*-citronellol, geraniol, citronellol, and linalool, consistent with the chemotype commonly reported for this species. The composition observed in the present study aligns closely with that described by ĆavarZeljković et al., who demonstrated that *G. macrorrhizum* typically accumulates high levels of citronellol- and geraniol-type monoterpenoids, with ploidy level and environmental factors exerting only minor quantitative effects [43]. Radulović et al. similarly identified *β*-citronellol and geraniol as the predominant constituents of Serbian populations, together with citronellyl esters and low amounts of sesquiterpene hydrocarbons—trends highly consistent with our findings [51]. Comparative analyses by Kremer et al. further confirmed the chemical stability of this chemotype across closely related taxa, showing that *G. macrorrhizum* possesses a distinctive micromorphological and phytochemical profile enriched in highly oxygenated monoterpenes, particularly in comparison with *G. dalmaticum* [52]. Sharopov et al. likewise reported analogous compositional patterns, underscoring the dominance of citronellol-type monoterpenoids and their limited ecological variability [32]. Broader comparative studies within the genus have highlighted substantial chemotaxonomic diversity among *Geranium* species, with taxa such as *G. phaeum* and *G. purpureum* exhibiting more sesquiterpene-rich profiles, whereas *G. macrorrhizum* consistently remains monoterpenol-dominant [9,53]. The biological relevance of its major constituents is well established, as citronellol and geraniol are widely recognized for their antioxidant, antimicrobial, and anti-inflammatory properties, as highlighted in recent reviews [33].

Taken together, these findings indicate that, although both species share a dominance of oxygenated monoterpenes, they display distinct quantitative patterns reflecting species-specific biosynthetic regulation and environmental influences. These differences support their chemotaxonomic differentiation within the *Geraniaceae* family.

### 3.3. Phenolic Composition of Pelargonium radula and Geranium macrorrhizum Extracts

The phenolic composition of *Pelargonium radula* has been poorly investigated, with previous studies concentrating primarily on its volatile constituents and the antimicrobial activity of selected flavonoids [24]. To date, no comprehensive profiling of its non-volatile secondary metabolites has been reported, and the phytochemical knowledge of this species remains substantially more limited than that of other *Pelargonium* taxa, such as *P. graveolens* and *P. sidoides* [54,55,56,57]. The present work, therefore, provides the first detailed HPLC-based characterization of phenolic acids and flavonoids in the ethanolic extract of *P. radula*, offering new chemical insights into this understudied species.

The extract exhibited a relatively simple phenolic profile, dominated by hydroxybenzoic and hydroxycinnamic acids, with only very low levels of flavonoids. The predominance of protocatechuic and ferulic acids, combined with moderate amounts of rosmarinic and vanillic acids, contrasts sharply with the richer phenolic composition typically reported for other *Pelargonium* species. For example, *P. graveolens* commonly accumulates substantial amounts of rosmarinic, caffeic, and *p*-coumaric acids, along with quercetin and rutin derivatives [54,58,59]. Similarly, the broader polyphenolic spectrum described for various *Pelargonium* species by Iancu et al. (2016) includes abundant flavonoids and catechins, compounds completely absent from the *P. radula* extract in the present study [57]. The lack of flavan-3-ols, together with the low amounts of rutin and hesperidin, supports the view that *P. radula* has comparatively weaker biosynthetic activity in the flavonoid branch of the phenylpropanoid pathway.

These interspecific differences are in line with previous research showing substantial variability in phenylpropanoid metabolism within the genus, often attributed to species-specific ecological adaptation or chemotaxonomic divergence [55,56,57,60]. The limited phenolic accumulation observed here is also consistent with earlier work indicating that *P. radula* has a lowerantioxidant potential than other *Pelargonium* species [54,57]. Moreover, the modest levels of rosmarinic and caffeic acid derivatives, in compared with the much higher concentrations typically recorded for *P. graveolens* or *P. odoratissimum*, support the classification of *P. radula* as a distinct phenolic chemotype [54,58].

The presence of simple hydroxybenzoic acids, including protocatechuic, gallic, and vanillic acids, even at low concentrations, aligns with previous findings for the genus [57]. However, the overall low phenolic richness and the absence of catechin-like compounds clearly differentiate *P. radula* from polyphenol-rich species known for their potent antioxidant and pharmacological potential. This also suggests that the biological properties of *P. radula* may rely more heavily on its essential-oil components, particularly citronellol- and geraniol-rich volatiles, than on its polyphenolic content, unlike species such as *P. graveolens* for which polyphenols play a central role.

In summary, the phenolic profile elucidated in this study substantially expands the current phytochemical understanding of *P. radula* and highlights its unique position within the genus. The extract’s limited diversity and relatively low phenolic content underscore species-specific metabolic traits and emphasize the need for further biochemical and ecological investigations into the regulation of phenolic biosynthesis in this taxon.

The phenolic composition of *Geranium macrorrhizum* has been explored to a certain extent in previous phytochemical studies, particularly in relation to its documented antioxidant and antimicrobial potential.Earlier studies on *G. macrorrhizum* and related taxa have reported the presence of polyphenols and general antioxidant capacity. Yet, most available data describe only total phenolic content or a narrow selection of marker compounds, without providing detailed chromatographic profiles [28,32,61]. More comprehensive phenolic investigations have been conducted for other *Geranium* species, such as *G. dalmaticum*, *G. purpureum*, *G. sibiricum*, and *G. carolinianum*, all of which show considerable interspecies variability in their polyphenolic patterns [61,62,63,64,65]. In this context, the present study provides, to date, the HPLC-based characterization of individual phenolic acids and flavonoids in *G. macrorrhizum*.

The extract exhibited a chemically richer profile than that of *Pelargonium radula*, with high levels of both hydroxybenzoic and hydroxycinnamic acids, as well as some flavonoid representatives. The predominance of rosmarinic acid and *p*-coumaric acid aligns well with previous reports describing hydroxycinnamate derivatives as characteristic constituents of *Geranium* spp. [61,62,63]. However, the notably high salicylic acid content observed in our extract is particularly noteworthy. Available literature does not report endogenous salicylic acid in *G. macrorrhizum*, and it reports only exogenous salicylic acid application to *Pelargonium zonale* in horticultural contexts [66]. Therefore, its detection here may reflect a previously unrecognized species-specific metabolic feature or an environmentally induced accumulation. This finding highlights the need for further focused investigations into the regulation of salicylate biosynthesis within the *Geraniaceae*.

The flavonoid profile, including (+)-catechin, (−)-epicatechin, rutin, and quercetin, corresponds with data reported for several *Geranium* species and supports the notion of an active flavonoid biosynthetic pathway in *G. macrorrhizum*. Catechin-type compounds, which were absent in *P. radula*, further emphasize the phytochemical divergence between the two taxa. The detection of kaempferol below the quantification threshold agrees with reports indicating that kaempferol glycosides are more typical for species such as *G. sibiricum* rather than *G. macrorrhizum* [64]. This pattern reinforces interspecific differentiation in the flavonoid branches of the phenylpropanoid pathway.

Overall, the phenolic richness of *G. macrorrhizum* aligns strongly with its documented biological potential, particularly regarding antioxidant and antimicrobial activities [32,61,62]. The combined presence of rosmarinic acid, *p*-coumaric acid, flavan-3-ols, and hydroxybenzoic acids provides a biochemical basis for these properties and places *G. macrorrhizum* among the more polyphenol-abundant representatives of the *Geraniaceae*. These findings significantly refine current phytochemical knowledge of the species and provide a robust foundation for future chemotaxonomic, ecological, and pharmacological investigations.

A direct comparison of the two investigated species reveals both shared and distinct phytochemical characteristics. In terms of volatile composition, both *Pelargonium radula* and *Geranium macrorrhizum* are dominated by oxygenated monoterpenes, particularly citronellol- and geraniol-type compounds, confirming their classification as monoterpenol-rich chemotypes. However, *P. radula* exhibits a more balanced citronellol/geraniol profile with moderate proportions of monoterpene esters and ketones, whereas *G. macrorrhizum* is characterized by a pronounced predominance of *β*-citronellol and a higher abundance of esterified derivatives. Regarding the non-volatile fraction, the two species differ markedly in their phenolic profiles. *P. radula* shows a relatively simple composition dominated by protocatechuic and ferulic acids with very low flavonoid content, while *G. macrorrhizum* displays substantially higher phenolic diversity and abundance, particularly in salicylic, rosmarinic, and *p*-coumaric acids, as well as catechin-type flavonoids. These differences reflect species-specific metabolic regulation within the *Geraniaceae* and underpin their divergent pharmacological potential. Overall, the combined volatile and non-volatile profiles highlight both chemotaxonomic relatedness and pronounced interspecific differentiation.

Although the present study is primarily descriptive, the comparative evaluation of the volatile and non-volatile fractions reveals clear interspecific patterns. Both species share a dominance of oxygenated monoterpenes; however, they differ markedly in their quantitative distribution and phenolic composition. The higher abundance of phenolic acids and flavan-3-ols in *Geranium macrorrhizum* suggests a stronger contribution of the phenylpropanoid pathway compared to *Pelargonium radula*, whose biological activity appears to rely predominantly on monoterpenoid constituents. The concurrent occurrence of citronellol-richess ential oil sand phenolic-rich extracts in *G. Macrorrhizum* may indicate complementary metabolic strategies that contribute to pharmacological potential. Nevertheless, due to the absence of inferential statistical analysis, the observed relationships should be interpreted cautiously and require further validation in future studies.

## 4. Materials and Methods

### 4.1. Plant Material

Aerial parts of *Pelargonium radula* and *Geranium macrorrhizum* were collected during the flowering season in the region of Gabrovo (Central Bulgaria). The species were identified by Assist. Prof. Petya Boycheva, from the Department of Biology, Faculty of Pharmacy at the Medical University of Varna (Bulgaria), and details on the collection conditions are summarized in Table 5.

The collected plant material was air-dried at room temperature in a shaded, well-ventilated space until it reached a constant weight. The dried samples were ground to about 0.5 mm particle size using a laboratory mill and then used for essential oil distillation and extract preparation.

### 4.2. Isolation of Essential Oils

Hydrodistillation was carried out in a Clevenger-type apparatus for 3 h. For each run, 100 g of powdered aerial parts were placed in a 2 L round-bottom flask with distilled water. The obtained essential oils were collected and stored in sealed vials at 4 °C until analysis. Oil yields were calculated as a percentage of the dry plant material weight (*v*/*w*, %).

### 4.3. Preparation of Dry Extracts

Powdered dry plant material (5.0 g) was extracted with 100 mL of 70% (*v*/*v*) ethanol in an Erlenmeyer flask, corresponding to a concentration of 50 mg/mL (5% *w*/*v*). The mixture was heated for two hours under continuous stirring on a magnetic stirrer with a reflux condenser to prevent solvent loss. After heating, the samples were subjected to ultrasonic extraction in a water bath sonicator for 30 min at room temperature. The extracts were cooled, stored at 4 °C for 48 h, and subsequently filtered through standard paper filters. In the end, the resulting extract was vacuum-concentrated (BUCHI R-300, Rotavapor, 50 °C and 97 mbar) to obtain the dried extract (DE), which was stored at −10 °C before chromatographic analysis.

### 4.4. Chemicals and Reagents

A homologous series of n-alkanes (C8–C30; Merck KGaA, Darmstadt, Germany) was used to determine retention indices (RI) for the GC–MS analysis. Essential oil samples were prepared by dilution with hexane (Sigma-Aldrich, Steinheim, Germany). All analytical standards for HPLC analysis were obtained from Sigma-Aldrich (Steinheim, Germany). Other solvents were of analytical grade and purchased from certified distributors.

### 4.5. GC-MS Analysis

The chemical composition of the essential oils (EOs) was analyzed using gas chromatography (GC) with an Agilent 7890A gas chromatograph (Agilent, Santa Clara, CA, USA) equipped with an HP-5 ms column (30 m × 250 µm × 0.25 µm). The temperature program was set as follows: 35 °C for 3 min, then increased at 5 °C per minute until reaching 250 °C, where it was held for 3 min, resulting in a total run time of 49 min. Helium served as the carrier gas at a constant flow rate of 1 mL/min, with a split ratio of 30:1. For GC/MS analysis, an Agilent 5975C mass spectrometer (Agilent) was used, also employing helium as the carrier gas. The same column and temperature program were applied as in the GC analysis. Compounds were identified by comparing their retention times and mass spectra with library data. The identified components were listed according to their retention time, and their relative abundances were expressed as percentages [67].

### 4.6. HPLC Analysis of Dry Extract

The ethanolic extracts (5% *w*/*v*) were analyzed by High-Performance Liquid Chromatography (HPLC), adapted from Krasteva et al. (2022) [68]. The analysis was performed on a Waters 1525 Binary Pump system (Waters, Milford, MA, USA) combined with a Waters 2484 Dual Absorbance Detector and Breeze 3.30 software, using a Supelco Discovery HS C18 column (5 µm, 250 × 4.6 mm). Before injection, extracts were filtered through a 0.25 µm membrane filter. Each injection used 20 µL at a flow rate of 1.0 mL/min. Gradient elution was carried out with solvent A (1% acetic acid in water) and solvent B (methanol). The gradient was as follows: 0–36 min, A 90–78%; 36–37 min, A 70%; 37–47 min, A 60%; 47–58 min, A 54%; 58–59 min, A 40%; 59–71 min, A 20%; 71–72 min, A 90%, maintained until 75 min. Detection occurred at 280 nm for gallic acid, protocatechuic acid, (+)-catechin, vanillic acid, syringic acid, (−)-epicatechin, *p*-coumaric acid, salicylic acid, and hesperidin, and at 360 nm for chlorogenic acid, caffeic acid, ferulic acid, rutin, rosmarinic acid, quercetin, and kaempferol. Quantification was performed using calibration curves of standard solutions with concentrations ranging from 10 to 500 µg/mL. The total run time of the HPLC analysis was 75 min.

### 4.7. Statistical Analysis

The presented results are based on independent analytical determinations and are reported as average values. Due to the descriptive nature of the study, no inferential statistical tests were performed.

## 5. Conclusions

This study provides the first integrated phytochemical comparison of *Pelargonium radula* and *Geranium macrorrhizum* cultivated in Bulgaria, combining GC–MS analysis of essential oils with HPLC profiling of phenolic acids and flavonoids. The two species displayed clearly distinct chemical signatures. *P. radula* showed a citronellol/geraniol-based essential oil and a relatively simple phenolic profile dominated by hydroxybenzoic and hydroxycinnamic acids with minimal flavonoid content. In contrast, *G. macrorrhizum* exhibited a *β*-citronellol–rich essential oil and a substantially more abundant non-volatile fraction, marked by high levels of salicylic, rosmarinic, and *p*-coumaric acids, as well as catechin-type flavonoids.

These findings expand the current phytochemical knowledge of both taxa, particularly the underexplored *P. radula*, and emphasize pronounced interspecific differences in phenylpropanoid metabolism within the *Geraniaceae*. The combined volatile and non-volatile profiles provide a robust foundation for future studies aimed at elucidating chemotaxonomic relationships, ecological adaptations, and potential pharmacological relevance. Further research into the biological activities of key constituents, especially the unexpectedly high salicylic acid content in *Geranium macrorrhizum*, is warranted to clarify their therapeutic potential and inform optimized selection of plant material for pharmacological applications.

Overall, the results indicate that *Geranium macrorrhizum* represents a more promising source of antioxidant and anti-inflammatory compounds, whereas *Pelargonium radula* shows greater potential for antimicrobial applications.

Future studies including larger sample sizes and inferential statistical analyses, are required to validate the observed interspecific relationships. By combining phytochemical profiling with botanical and ethnomedicinal context, this study provides an integrated framework for understanding the biological potential of both species.

## 6. Limitations and Future Perspectives

The present study has several limitations that should be acknowledged. First, the investigation was primarily descriptive and based on phytochemical profiling, without applying inferential statistical analyses. In addition, the analyses were conducted on samples collected from a single geographical region and during a limited harvesting period, which may not fully reflect seasonal or environmental variability. Furthermore, the pharmacological potential of the investigated species was inferred mainly from their chemical composition and literature data, without direct biological activity assays.

Future research should include multi-seasonal and multi-regional sampling, larger datasets, and comprehensive statistical evaluations. In addition, in vitro and in vivo pharmacological studies are required to validate the biological relevance of the identified compounds. Integrative approaches combining metabolomics, genomics, and bioactivity-guided fractionation would further deepen understanding of the therapeutic potential of *Pelargonium radula* and *Geranium macrorrhizum*.

## Figures and Tables

**Table 1:** Chemical composition of the essential oil from *Pelargonium radula*.

Compound	^1^ Relative Retention Index	% of ^2^ TIC	Class of
			Compound
(3*Z*)-Hexenol	851	0.56	^3^ MO
*α*-Thujene	925	0.11	^4^ MH
*α*-Pinene	933	0.34	MH
Sabinene	970	0.17	MH
*β*-Pinene	975	0.22	MH
*β*-Myrcene	989	0.21	MH
*δ*-2-Carene	1002	0.13	MH
*α*-Phellandrene	1003	0.14	MH
*p*-Cymene	1021	0.11	MH
Limonene	1025	0.22	MH
Eucalyptol	1027	0.10	MO
(*Z*)-*β*-Ocimene	1033	0.15	MH
(*E*)-*β*-Ocimene	1045	0.12	MH
*cis*-Linalool oxide	1068	0.14	MO
*trans*-Linalool oxide	1085	0.16	MO
Linalool	1096	6.07	MO
*cis*-Rose oxide	1107	0.34	MO
Phenyl ethyl alcohol	1108	0.08	^5^ O
*trans*-Rose oxide	1123	0.24	MO
Menthone	1149	0.26	MO
Isomenthone	1159	5.75	MO
Menthol	1168	0.15	MO
Isomenthol	1180	0.27	MO
*α*-Terpineol	1187	0.34	MO
Citronellol	1224	28.06	MO
Nerol	1227	1.23	MO
Neral	1236	0.60	MO
Geraniol	1250	25.05	MO
Geranial	1265	1.01	MO
Citronellylformate	1272	7.30	MO
Nerylformate	1281	0.12	MO
Geranyl formate	1299	3.38	MO
Citronellic acid	1313	0.15	MO
*α*-Cubebene	1349	0.11	^6^ SH
Citronellyl acetate	1351	0.34	MO
*α*-Copaene	1375	0.22	SH
Geranyl acetate	1380	0.37	MO
*β*-Bourbonene	1388	0.45	SH
*β*-Caryophyllene	1418	0.56	SH
*β*-Copaene	1431	0.11	SH
Aromadendrene	1440	0.26	SH
Citronellyl propanoate	1445	0.34	MO
*α*-Humulene	1453	0.29	SH
*allo*-Aromadendrene	1459	0.20	SH
Geranyl propanoate	1477	0.18	MO
Germacrene D	1481	1.29	SH
Viridiflorene	1497	0.66	SH
*γ*-Cadinene	1515	0.14	SH
Geranyl isobutanoate	1516	0.11	MO
*δ*-Cadinene	1524	0.56	SH
Geranyl butanoate	1564	0.16	MO
Caryophyllene oxide	1584	0.21	^7^ SO
2-Phenylethyl tiglate	1586	1.24	O
10-*epi*-*γ*-Eudesmol	1624	4.24	SO
1-*epi*-Cubenol	1629	0.19	SO
*γ*-Eudesmol	1632	0.22	SO
Cubenol	1647	0.56	SO
*α*-Cadinol	1654	0.79	SO
Citronellyltiglate	1668	0.34	MO
Geranyl tiglate	1698	2.13	MO

^1^ RI—retention index; ^2^ TIC—total ion chromatogram; ^3^ MO—oxygenated monoterpenes; ^4^ MH—monoterpene hydrocarbons; ^5^ O—other non-terpenoid compounds ^6^ SH—sesquiterpene hydrocarbons; ^7^ SO—oxygenated sesquiterpenes.

**Table 2:** Chemical composition of the essential oil from *Geranium macrorrhizum*.

Compound	^1^ Relative Retention Index	% of ^2^ TIC	Class of
			Compound
*α*-pinene	940	0.56	^3^ MH
*β*-myrcene	991	0.14	MH
*α*-phellandrene	1003	0.22	MH
*p*-cymene	1025	0.17	MH
limonene	1030	0.11	MH
*cis*-rose oxide	1109	1.35	^4^ MO
*trans*-rose oxide	1126	0.45	MO
linalool	1097	4.03	MO
menthol	1172	7.53	MO
iso-menthol	1183	0.22	MO
*α*-terpineol	1189	0.34	MO
*β*-citronellol	1226	31.37	MO
geraniol	1253	14.79	MO
citronellal	1154	0.11	MO
neral	1239	0.67	MO
geranial	1268	0.90	MO
iso-menthone	1163	1.24	MO
citronellylformate	1274	7.55	MO
geraniol formate	1299	2.60	MO
citronellyl acetate	1353	1.24	MO
citronellyl propionate	1447	0.22	MO
citronellyl pentanoate	1627	4.74	MO
geranyl valerate	1658	0.79	MO
geranyl tiglate	1698	1.12	MO
*α*-cubebene	1352	0.22	^5^ SH
*α*-copaene	1377	0.79	SH
*β*-bourbonene	1389	0.55	SH
*β*-caryophyllene	1420	1.80	SH
aromadendrene	1442	0.90	SH
amorpha-4,11-diene	1452	0.67	SH
*α*-humulene	1455	0.34	SH
allo-aromadendrene	1461	0.23	SH
*cis*-muurola-4(14),5-diene	1467	0.79	SH
*γ*-muurolene	1480	0.34	SH
germacrene D	1482	2.47	SH
*α*-amorphene	1485	0.16	SH
*β*-selinene	1491	0.67	SH
trans-muurola-4(14),5-diene	1494	0.90	SH
*α*-muurolene	1502	0.26	SH
(E,E)-*α*-farnesene	1507	0.12	SH
*β*-bisabolene	1507	0.45	SH
*γ*-cadinene	1515	1.20	SH
*trans*-calamenene	1524	0.67	SH
*δ*-cadinene	1525	0.90	SH
*trans*-cadina-1,4-diene	1536	0.56	SH
spathulenol	1580	0.24	^6^ SO
1-epi-cubenol	1630	0.20	SO
cubenol	1648	0.22	SO
*α*-muurolol	1648	0.30	SO
*α*-cadinol	1656	0.34	SO
2-phenyl ethyl tiglate	1587	1.02	^7^ O

^1^ RI—retention index; ^2^ TIC—total ion chromatogram; ^3^ MH—monoterpene hydrocarbons; ^4^ MO—oxygenated monoterpens; ^5^ SH—sesquiterpene hydrocarbons; ^6^ SO—oxygenated sesquiterpenes; ^7^ O—other non-terpenoid compounds.

**Table 3:** HPLC Profile of Phenolic Acids and Flavonoids in the Ethanolic Extract of *Pelargonium radula*.

Type of Phenols	Compounds	Content, mg/g DE *
	Gallic acid	0.41
	Protocatehuic acid	3.71
	Chlorogenic acid	NF **
	Vanillic acid	1.03
	Caffeic acid	0.17
Phenolic acids	Syringic acid	NF
	*p*-Coumaric acid	ULOQ ***
	Ferulic acid	3.17
	Salicylic acid	NF
	Rosmarinic acid	1.66
	(+)-Catechin	NF
	(−)-Epicatechin	NF
Flavonoids	Rutin	2.51
	Hesperidin	0.01
	Quercetin	ULOQ
	Kaempherol	ULOQ

* mg/g DE—milligram per gram dry extract; ** NF—not found; *** ULOQ—under the limit of quantification.

**Table 4:** HPLC Profile of Phenolic Acids and Flavonoids in the Ethanolic Extract of *Geranium macrorrhizum*.

Type of Phenols	Compounds	Content, mg/g DE *
	Gallic acid	2.05
	Protocatehuic acid	0.77
	Chlorogenic acid	0.96
	Vanillic acid	7.64
	Caffeic acid	NF **
Phenolic acids	Syringic acid	3.23
	*p*-Coumaric acid	10.60
	Ferulic acid	2.21
	Salicylic acid	37.33
	Rosmarinic acid	18.31
	(+)-Catechin	2.43
	(−)-Epicatechin	3.17
Flavonoids	Rutin	5.03
	Hesperidin	NF
	Quercetin	1.23
	Kaempherol	ULOQ ***

* mg/g DE—milligram per gram dry extract; ** NF—not found; *** ULOQ—under the limit of quantification.

**Table 5:** Plant materials used in the study.

Species	Locality (Region)	GPS Coordinates	Date of Collection	Plant Part Used
*Pelargonium radula*	Gabrovo, Central Bulgaria	42°52′ N, 25°20′ E	June 2025	Aerial parts
*Geranium macrorrhizum*	Gabrovo, Central Bulgaria	42°52′ N, 25°20′ E	June 2025	Aerial parts

## Data Availability

The original contributions presented in this study are included in the article. Further inquiries can be directed to the corresponding author.

## Supplementary Materials

The following supporting information can be downloaded at: https://www.mdpi.com/article/10.3390/ph19030346/s1 Figure S1: GC-MS chromatogram of essential oil, obtained from *Pelargonium radula*; Figure S2: GC-MS chromatogram of essential oil, obtained from *Geranium macrorrhizum*; Figure S3: HPLC chromatograms of ethanolic extract of *Pelargonium radula*. HPLC fingerprints of SAMPLE 1 (A and C) and STANDARDS (B and D) at 280 nm (A, B) and 340 nm (C, D): 1—Gallic acid; 2—Protocatehuic acid; 3—(+)-Catechin; 4—Chlorogenic acid; 5—Vanillic acid; 6—Caffeic acid; 7—Syringic acid; 8—(−)-Epicatechin; 9—*p*-Coumaric acid; 10—Ferulic acid; 11—Salicylic acid; 12—Rutin; 13—Hesperidin; 14—Rosmarinic acid; 15—Quercetin; 16—Kaempherol; Figure S4: HPLC chromatograms of ethanolic extract of *Geranium macrorrhizum*. HPLC fingerprints of SAMPLE 2 (A and C) and STANDARDS (B and D) at 280 nm (A, B) and 340 nm (C, D): 1—Gallic acid; 2—Protocatehuic acid; 3—(+)-Catechin; 4—Chlorogenic acid; 5—Vanillic acid; 6—Caffeic acid; 7—Syringic acid; 8—(−)-Epicatechin; 9—*p*-Coumaric acid; 10—Ferulic acid; 11—Salicylic acid; 12—Rutin; 13—Hesperidin; 14—Rosmarinic acid; 15—Quercetin; 16—Kaempherol.

## Abbreviations

The following abbreviations are used in this manuscript:

GC-MS	Gas Chromatography coupled with Mass Spectrometry
HPLC	High-Performance Liquid Chromatography
DE	Dry extract
MH	Monoterpene hydrocarbons
MO	Oxygenated monoterpenes
NF	Not found
O	Other compounds
RI	Retention indices
SO	Oxygenated sesquiterpenes
SH	Sesquiterpene hydrocarbons
ULOQ	Under the limit of quantification
