# Supplementary material for: Comparative Phytochemical Analysis of the Aerial Parts of Pelargonium radula and Geranium macrorrhizum Cultivated in Bulgaria Using GC-MS and HPLC
Source: Pharmaceuticals (Basel). 2026 Feb 24;19(3):346. doi: 10.3390/ph19030346 (PMC13028625; doi:10.3390/ph19030346)
Supplement: Supplementary file 1 [file pharmaceuticals-19-00346-s001.zip › pharmaceuticals-4144924-supplementary.pdf]

Contents:

Figure S1. GC-MS chromatogram of essential oil, obtained from *Pelargonium radula*,

Figure S2. GC-MS chromatogram of essential oil, obtained from *Geranium macrorrhizum.*

Figure S3. HPLC chromatograms of ethanolic extract of *Pelargonium radula*. HPLC fingerprints of SAMPLE 1 (A and C) and STANDARDS (B and D) at 280 nm (A, B) and 340 nm (C, D): 1 - Gallic acid; 2 - Protocatehuic acid; 3 - (+)-Catechin; 4 - Chlorogenic acid; 5 - Vanillic acid; 6 - Caffeic acid; 7 - Syringic acid; 8 - (-)-Epicatechin; 9 - *p*-Coumaric acid; 10 - Ferulic acid; 11 - Salicylic acid; 12 – Rutin; 13 – Hesperidin; 14 - Rosmarinic acid; 15 - Quercetin; 16 - Kaempherol.

Figure S4. HPLC chromatograms of ethanolic extract of *Geranium macrorrhizum*. HPLC fingerprints of SAMPLE 2 (A and C) and STANDARDS (B and D) at 280 nm (A, B) and 340 nm (C, D): 1 - Gallic acid; 2 - Protocatehuic acid; 3 - (+)-Catechin; 4 - Chlorogenic acid; 5 - Vanillic acid; 6 - Caffeic acid; 7 - Syringic acid; 8 - (-)-Epicatechin; 9 - *p*-Coumaric acid; 10 - Ferulic acid; 11 - Salicylic acid; 12 – Rutin; 13 – Hesperidin; 14 - Rosmarinic acid; 15 – Quercetin; 16 - Kaempherol.

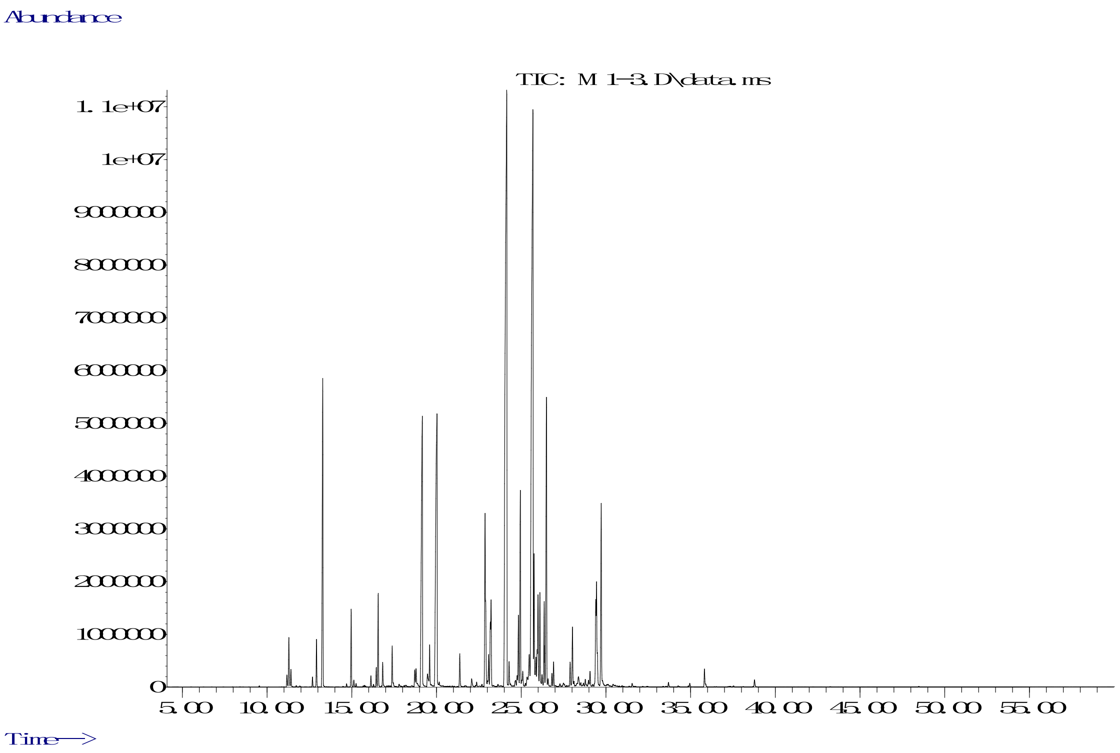

Figure S1. GC-MS chromatogram of essential oil, obtained from *Pelargonium radula,*

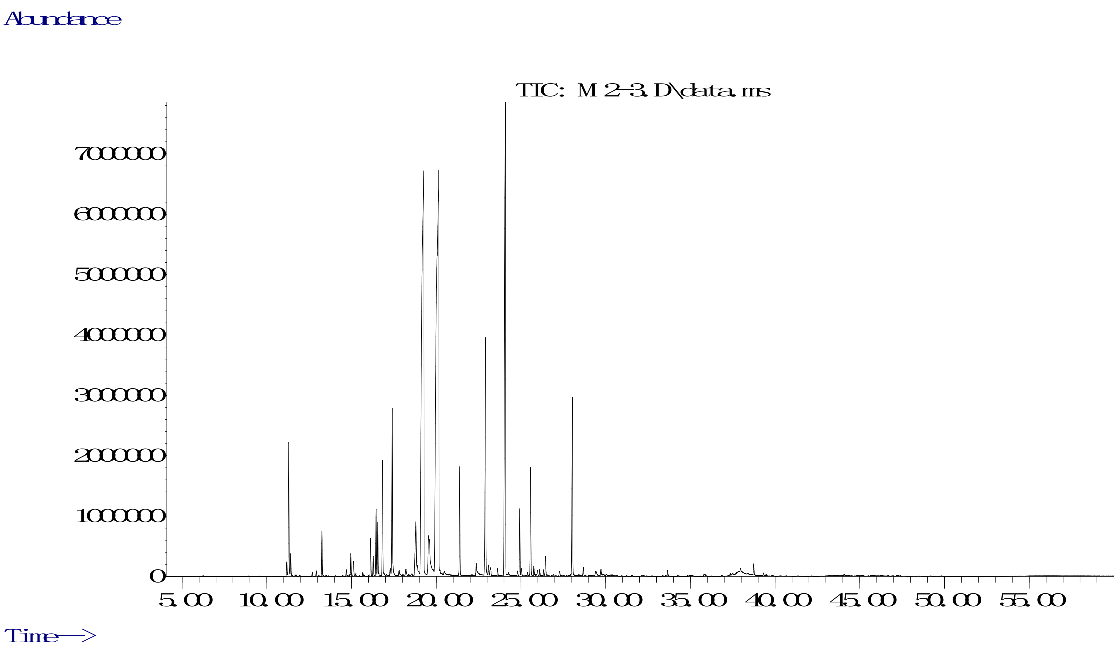

Figure S2. GC-MS chromatogram of essential oil, obtained from *Geranium macrorrhizum.*

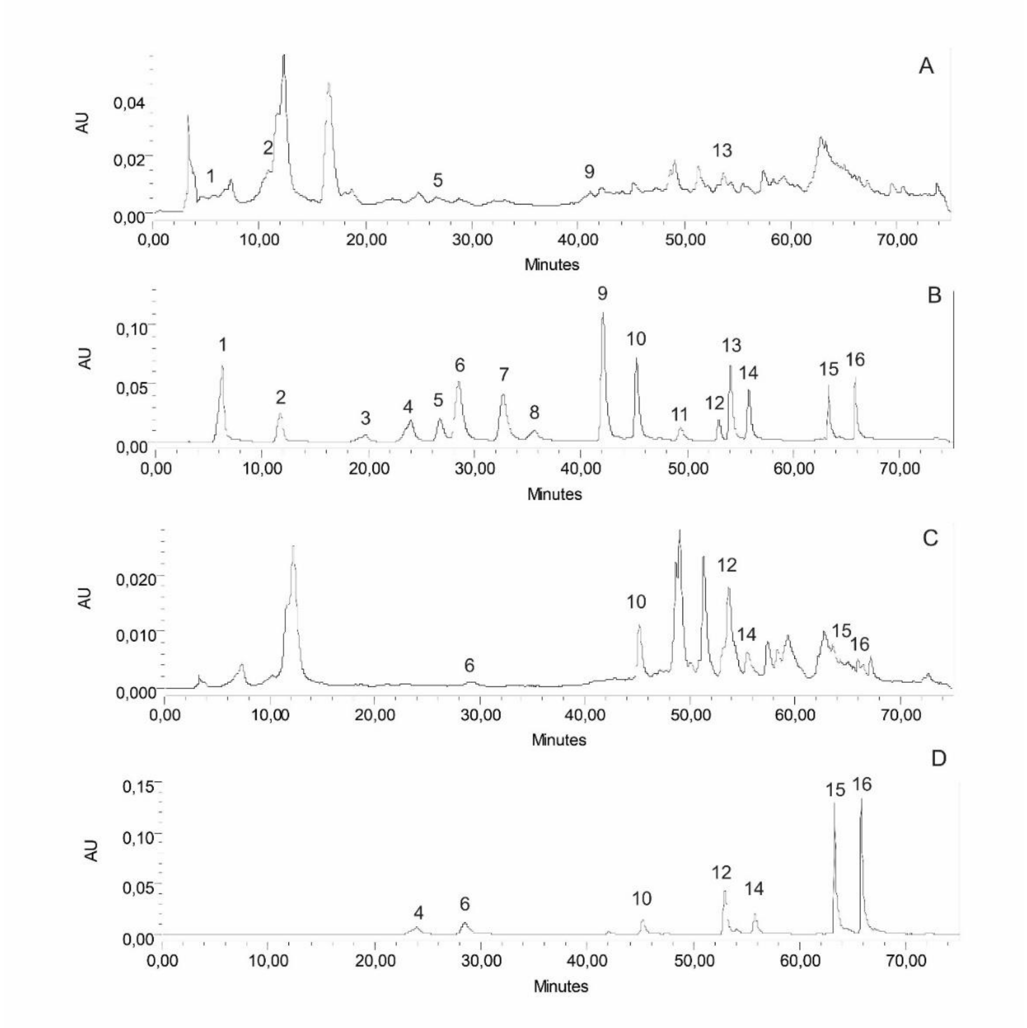

Figure S3. HPLC chromatograms of ethanolic extract of *Pelargonium radula*. HPLC fingerprints of SAMPLE 1 (A and C) and STANDARDS (B and D) at 280 nm (A, B) and 340 nm (C, D): 1 - Gallic acid; 2 - Protocatehuic acid; 3 - (+)-Catechin; 4 - Chlorogenic acid; 5 - Vanillic acid; 6 - Caffeic acid; 7 - Syringic acid; 8 - (-)-Epicatechin; 9 - *p*-Coumaric acid; 10 - Ferulic acid; 11 - Salicylic acid; 12 – Rutin; 13 – Hesperidin; 14 - Rosmarinic acid; 15 - Quercetin; 16 - Kaempherol.

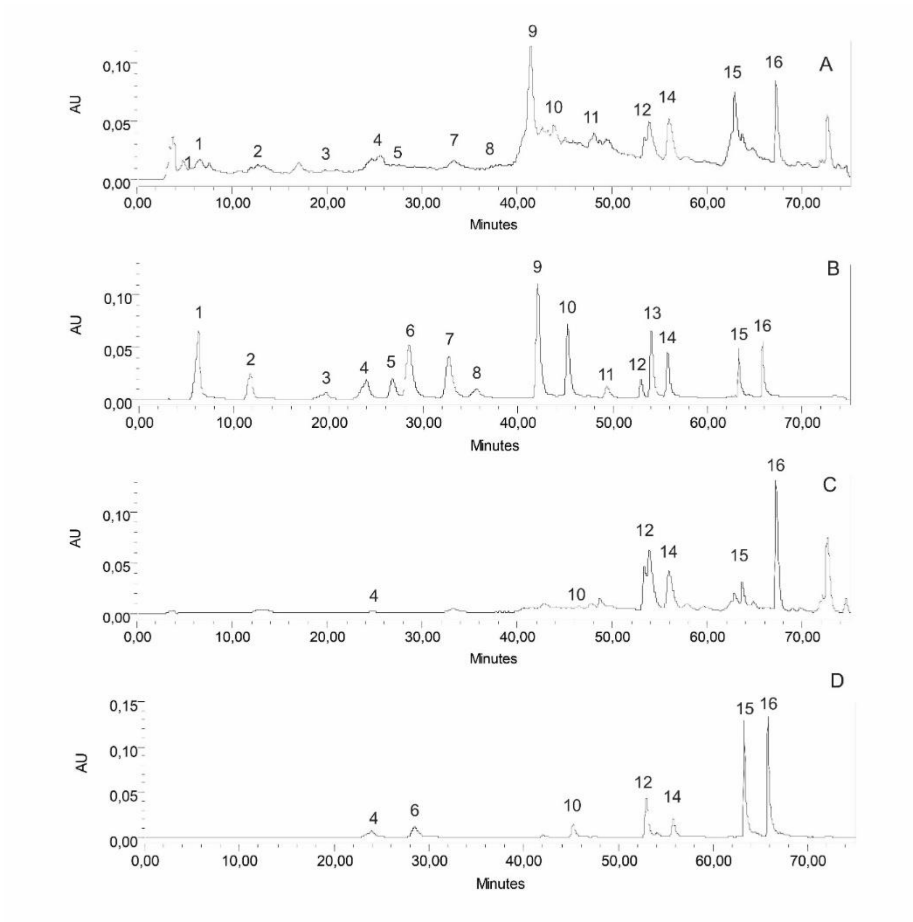

Figure S4. HPLC chromatograms of ethanolic extract of *Geranium macrorrhizum*. HPLC fingerprints of SAMPLE 2 (A and C) and STANDARDS (B and D) at 280 nm (A, B) and 340 nm (C, D): 1 - Gallic acid; 2 - Protocatehuic acid; 3 - (+)-Catechin; 4 - Chlorogenic acid; 5 - Vanillic acid; 6 - Caffeic acid; 7 - Syringic acid; 8 - (-)-Epicatechin; 9 - *p*-Coumaric acid; 10 - Ferulic acid; 11 - Salicylic acid; 12 – Rutin; 13 – Hesperidin; 14 - Rosmarinic acid; 15 – Quercetin; 16 - Kaempherol.
